# Alpha-1-antitrypsin suppresses oxidative stress in preeclampsia by inhibiting the p38MAPK signaling pathway: An in vivo and in vitro study

**DOI:** 10.1371/journal.pone.0173711

**Published:** 2017-03-30

**Authors:** Ya-Ling Feng, Yong-Xiang Yin, Jian Ding, Hua Yuan, Lan Yang, Jian-Juan Xu, Ling-Qin Hu

**Affiliations:** 1 Department of Obstetrics and Gynecology, Wuxi Matemal and Child Health Hospital Affiliated to Nanjing Medical University, Wuxi, P.R. China; 2 Department of Pathology, The Affiliated Maternity and Child Health Hospital of Nanjing Medical University, Wuxi, P.R. China; University of PECS Medical School, HUNGARY

## Abstract

This present study was designed to investigate the effects of alpha-1-antitrypsin (AAT) on oxidative stress in preeclampsia (PE) by regulating p38 mitogen-activated protein kinase (p38MAPK) signaling pathway. HTR8/SVneo cells were randomly assigned into normal, hypoxia/reoxygenation (H/R), HR + AAT and HR + siRNA-AAT groups. Quantitative real-time polymerase chain reaction (qRT-PCR) and Western blotting were used to detect the mRNA and protein expressions of p-p38MAPK, AAT, signal transducer and activator of transcription 1 (STAT1) and activating transcription factor2 (ATF2). Flow cytometry, scratch test, cell counting kit-8 (CCK-8) assay and the 3-(4,5)-dimethylthiazol (-z-y1)-3,5-di- phenyltetrazolium bromide (MTT) assay were conducted to detect reactive oxygen species (ROS) and cell apoptosis, cell migration, proliferation and cytotoxicity, respectively. Mouse models in PE were established, which were divided into normal pregnancy (NP), PE and PE + AAT groups with blood pressure and urine protein measured. Chromatin immunoprecipitation (ChIP) and enzyme-linked immunosorbent assay (ELISA) were conducted to detect the activity of oxidative stress-related kinases and expressions of inflammatory cytokines and coagulation-related factors in cells and mice placenta. Immunohistochemistry, Western blotting and terminal deoxynucleotidyl transferase-mediated dUTP nick end labeling (TUNEL) assay were performed to detect AAT and p38MAPK expressions, apoptosis-related protein expressions, and apoptosis rate in mice placenta. Compared with the normal group, the H/R group had decreased expression of AAT, activity of superoxide dismutase (SOD) and GSH-Px, cell proliferation and migration, but increased p38MAPK, STAT1, ATF2, MDA, H_2_O_2_, inflammatory cytokines, coagulation-related factors, cell cytotoxicity, ROS, apoptotic factors and apoptosis rate. Compared with the H/R group, the HR + ATT group had increased expressions of AAT, activity of SOD and GSH-Px, cell proliferation and migration but decreased p38MAPK, STAT1, ATF2, malonyldialdehyde (MDA), H_2_O_2_, inflammatory cytokines and coagulation-related factors, cell cytotoxicity, ROS, apoptotic factors and apoptosis rate, while opposite results were observed in the HR + siRNA-ATT group. Compared with the NP group, the PE group had decreased activity of SOD and GSH-Px but increased MDA, H_2_O_2_, AAT, p38MAPK, inflammatory cytokines, coagulation-related factors and apoptosis rate. The indexes in the PE + AAT group were between the NP and PE groups. Thus, we concluded that AAT suppressed oxidative stress in PE by inhibiting p38MAPK signaling pathway.

## Introduction

Preeclampsia (PE) is a specific disorder related to pregnancy with a manifestation of proteinuria, gestational hypertension, and an excessive inflammatory response, as well as systemic endothelial cell activation [1]. PE is the third major cause of maternal morbidity and mortality across the world and affects 3% to 5% of all pregnancies, with estimated 60,000 maternal deaths each year worldwide [2]. Pregnancy itself increases susceptibility to oxidative stress, possibly resulting in tissue damage [3]. However, the production of pro-oxidants and reactive oxygen species (ROS) towards the end of pregnancy is increased in normal uncomplicated pregnancy and maintained through the accumulation of antioxidants, such as superoxide dismutase (SOD), tocopherols, and carotenoids, as well as ascorbic acid [4]. Interestingly, the pro-oxidant (MDA) and antioxidant (GSH and SOD) balance is disturbed in pregnancy with PE, and increased oxidative stress along with antioxidative defense mechanisms may contribute to the progression of PE [5].

P38 mitogen-activated protein kinase (p38MAPK), a kinase belonging to the MAPK superfamily, which modulates diverse cellular events, is of great importance for placental organogenesis along with trophoblastic growth and invasion [6]. It has been found that oxidative stress is an important inducer of the synthesis of the placenta along with the release of pro-inflammatory factors, and most of these effects are regulated by the p38MAPK signaling pathway [7]. Alpha-1-antitrypsin (AAT), a serine protease inhibitor (serpins) encoded by the AAT gene that includes seven exons and six introns, is located on chromosome 14 at q31 within the serpin-related gene cluster [8]. AAT, an abundant protein that is mainly expressed in hepatocytes, primarily functions to inhibit the destructive neutrophil proteases elastase, and proteinase 3, as well as cathepsin G [9]. It has been suggested that accumulation of AAT weakens neutrophil infiltration during acute and chronic inflammation and decreases neutrophil infiltration into the kidney during the attack of ischemia-reperfusion [10]. A deficiency of AAT will lead to misfolded mutant protein expression, which is further involved in some disorders, such as tissue injury and oxidative stress [11]. A recent study suggests that AAT injection can not only inhibit oxidative stress but also inactivates the PAK/signal transducer and activator of transcription 1 (STAT1)/p38 signaling pathway in PE [12]. As previous researches were mainly focused on the vascular endothelial cells and tissues or the relationship between AAT or p38MAPK signaling pathway and the development of PE, there was no study conducted regarding how AAT affects the development of PE through oxidative stress by regulating p38MAPK signaling pathway in trophoblast cells. Therefore, this current study aims to explore the role of AAT and the p38MAPK signaling pathway in oxidative stress during the onset of PE and to investigate whether AAT treatment could be therapeutically beneficial to the improvement of PE.

## Materials and methods

### Cell culture

The HTR-8/SVneo cell lines were obtained from Professor Charles H. Graham in Departments of Biomedical and Molecular Sciences and Urology, Queen's University, Canada. After resuscitation, the cells were cultured in 8 mL of 1640 medium (No. 10565018; Gibco, USA) containing 5% fetal bovine serum (FBS) (No. 10099141; Gibco, USA) and 5 μL of penicillin and streptomycin (No. 15140122; Gibco, USA). The pH value of the medium was 7.0–7.2, and the cells were cultured in 5% CO_2_ at 37°C. Cell growth was observed every day, with regular medium changes and cell subcultures.

### Plasmid construction

In cultured HTR8/SVneo cell lines, with human DNA as a template, the AAT gene sequence was amplified in primary HTR8/SVneo cell lines by quantitative real-time polymerase chain reaction (qRT-PCR). Then PcDNA3.1 (+) myc-B lentivirus vector was inserted to construct the AAT overexpression plasmid (sense: 5’-GTACCCTAAACCAGCCAGACAG-3’; antisense: 5’-GCTTCATCATAGGGACCTTCAC-3’). The siRNA-AAT, siRNA which interferes AAT, was designed, and its sequences are as follows: sense: 5’-AUGGUGGGCAGGGAAGACUGCUUCC-3’; antisense, 5’-GGAAGCAGUCUUC CCUGCCACCAU-3’. Then, the siRNA-AAT plasmids were transferred into the HTR-8/SVneo cell lines, and G418 was used to select HTR8/SVneo cell lines stably expressing AAT. Western blotting was used to determine whether AAT gene was over-expressed or inhibited. HTR8/SVneo cells with stable siRNA-AAT or AAT over-expression plasmids were obtained for the following study.

### Establishment of the HTR8/SVneo cell models of oxidative stress damage and grouping

Our preliminary experimental results showed that two hours of hypoxia, four hours of reoxygenation, and 50 μmol/L of exogenous AAT were the best conditions for AAT to function. The cells were grouped as follows: the normal group (HTR8/SVneo cells normally cultured without any H/R treatment), the hypoxia/reoxygenation (H/R) (HTR8/SVneo cells treated with 2 h hypoxia and 4 h reoxygenation), the HR + AAT group (HTR8/SVneo cells treated with 2 h hypoxia and 4 h reoxygenation after transfection with AAT over-expression plasmid) and the HR + siRNA-AAT group (HTR8/SVneo cells treated with 2 h hypoxia and 4 h reoxygenation after transfection with siRNA-AAT overexpression plasmid). The culture medium of each group was changed into serum-free medium, and then the cells were cultured for 24 h. The cells in the control group were cultured in a humidified incubator containing 95% air and 5% CO_2_ (20% O_2_) for 8 h, and the cells in the H/R group were first cultured in a tri-gas incubator containing 1% O_2_, 5% CO_2_ and 94% N_2_ for 2 h and later in an incubator (5% CO_2_, 20% O_2_) for 4 h.

### Quantitative Real-Time Polymerase Chain Reaction (qRT-PCR)

The total RNA of HTR8/SVneo cells was extracted using Trizol. RNA purity and concentration were measured by ultraviolet spectrophotometry, while its completeness was observed with agarose gel electrophoresis (AGE). Reverse transcription was achieved using the Primescript^™^ RT Reagent Kit (Takara Biotechnology Company, Liaoning, China) and qRT-PCR amplification was performed using the SYBR^®^ Premix Ex Taq^™^ Kit (Takara Biotechnology Company, Liaoning, China) with the following amplification conditions: pre-denaturation at 95°C for 3 min, and 40 cycles of 95°C for 30 s, 58°C for 30 s and 72°C for 4 s. The amplification primer sequences are listed in Table 1. The qPT-PCR results were analyzed using an OpticonMonitor3 (Bio-Rad Laboratories, Inc. CA, USA). For each reaction, the threshold cycle value (Ct) was determined by the threshold settled in the lowest point of logarithmic amplification curve, and the data were analyzed by 2^-ΔΔCt^, which represents the ratios of gene expressions between the experimental group and control group. The formula is as follows: ΔΔCt = [Ct(target gene)—Ct(reference gene)]experimental group—[Ct(target gene)–Ct(reference gene)]_control group_. These experiments were repeated 3 times in each group.

**Table 1:** The primer sequences for qRT-PCR.

Gene	Sequence
β-actin	F: 5'-TGCTGTCCCTGTATGCCTCTGG-3’
	R: 5'-TTTGATGTCACGCACGATTTCC-3’
P38MAPK	F: 5'-TCGAGACCGTTTCAGTCCAT-3'
	R: 5'-CCACGGACCAAATATCCACT-3'
Fas/FasL	F: 5'-GAATGCAAGGACTGATAGC-3'
	R: 5'-TGGTTCGTGTGCAAGGCTC-3'
Bcl-2	F: 5'-GGGACGCGAAGTGCTATTGGTA-3'
	R: 5'-CAGGCTGGAAGGAGAAGATGC-3'
Caspase	F: 5'-CAAACTTTTTCAGAGGGGATCG-3'
	R: 5'-GCATACTGTTTCAGCATGGCAC-3'
STAT1	F: 5'-GTTGAACCCTACACGAAG-3'
	R: 5'-CTACAGAGCCCACTATCC-3'
ATF2	F: 5'-CTCGAGAAAACAAGGACCTGTGGAAT-3'
	R: 5'-GGATCCTCCACTTCCTGAGGGCTG-3'

Note: AAT, alpha-1-antitrypsin; p38MAPK, p38 mitogen-activated protein kinase; STAT1, signal transducer and activator of transcription 1; ATF2, activating transcription factor 2; Bcl-2, B-cell lymphoma-2; qRT-PCR, quantitative real-time polymerase chain reaction; F, forward; R, reverse.

### Western blotting

The total protein was extracted from split HTR8/SVneo cells, 20 μg of which was mixed well with loading buffer and then processed by sodium dodecyl sulfate-polyacrylamide gel electrophoresis (SDS-PAGE) with a 10% separation gel/5% stacking gel. Subsequently, the protein was electrotransferred onto a cellulose nitrate membrane. After processing with 5% skimmed milk powder and phosphate buffer saline (PBS) solution and sealing for 1 h at room temperature. The membrane was then incubated with the primary antibodies (AAT: ab7633, 10 μg/mL; p38 MAPK: GTX103009, 1/1000; signal transducer and activator of transcription 1 (STAT1): ab31369, 1/1000; activating transcription factor2 (ATF2): b47476, 1/500; Fas: ab82419, 1/1000; FasL: ab15285, 1 μg/mL; B-cell lymphoma-2 (Bcl-2): ab32124, 1/1000; BCL2-associated X protein (Bax): ab53154, 1/500; Caspase-3: ab13847, 1/500; all from Abacam, Cambridge, MA, USA) overnight at room temperature. After 3 washes with PBS, the membrane was incubated for 1 h with a goat anti-rabbit IgG H&L secondary antibody (ab6702, 1/1000, Abacam, Cambridge, MA, USA) conjugated to horseradish peroxidase (HRP) at room temperature. After washing with PBS 3 times, the membrane was developed using enhanced chemiluminescence. At the same time, with β-actin (1: 5000, KC-5A08, Kangcheng Biology Engineering Co., Ltd. Shanghai, China) as a internal reference, the images were acquired using a Gel dol EZ Bio-Rad imaging instrument (GEL DOC EZ IMAGER, Bio-Rad, California, USA). The gray value of the target bands was calculated using Image J software. The experiment was repeated 3 times.

### Chromatin Immunoprecipitation (ChIP)

ChIP was undertaken according to the kit instructions. On the basis of well-prepared conditions (100W, ultrasound processing for 10 s per round, a total of 5 rounds with a 30 s intervals), the chromatin of the cells in the lysate was split and bound to protein to form protein-DNA complexes. The protein-DNA was centrifuged at 4°C, and the supernatant was obtained. Ultrasound-processed supernatant was added to protein G- agarose beads for pre-clearing and then centrifuged, after which 1% (v/v) of supernatant was reserved as the Input solution, and the rest of the supernatant was added to co-immunoprecipitation antibodies, including positive control antibody (0.1 μL/tube), negative control antibody (0.1 μL /tube) and target antibody (MDA antibody, T-SOD antibody, GSH-PX antibody, 10 μL/tube), and incubated at 4°C overnight. while shaking The immunoprecipitation of protein-DNA complexes was washed by elution buffer, and the same volume of protein-DNA was uncross-linked by elution buffer in a tube containing Input solution. The DNA was then released and recycled by DNA purification. A proper volume of dissolved DNA was selected as the template, and the viability of enzyme proteins by Western blotting. The superoxide dismutase (SOD) enzyme activity in the sample was equal to the activity unit of SOD in detection system, which was calculated by inhibition percentage /(1- inhibition percentage). The activity unit of SOD was converted to U/mg protein. Each experiment was repeated three times.

### Enzyme-Linked Immunosorbent Assay (ELISA)

Using 0.05 M, pH 9.6, carbonate-coated buffer, the antibody was diluted until the protein concentration was within 1–10 μg/ml. Then, 0.1 mL of diluted antibody solution was added to each well in a polystyrene plate and the plates were stored overnight at 4°C. Subsequently, the solution in each well was aspirated the next day, and the polystyrene board was washed by washing buffer for 3 times (3 min per time). Then 0.1 mL of diluted sample was added into the coated wells, and the plates were incubated at 37°C for 1 h. After washing again, 0.1 mL of diluted enzyme-labeled antibody was added into each well and the plates were incubated at 37°C for 0.5 to 1 h then washed, followed by the addition of 0.1 mL of tetramethylbenzidine (TMB) substrate to react at 37°C for 10~30 min for developing. After that, 0.05 mL of 2 M sulfuric acid was added to stop the developing reaction. The optical density (OD) value of each well was detected at a wavelength of 450 nm by ELISA after setting the blank wells as zero. It should be noted that the OD values were determined at 410 nm if 2, 2'-azinobis-3-ethylbenzotiazo-line-6-sulfonic acid (ABTS) was used for developing. If the detection result was 2.1 times larger than OD value of the negative control, it would be considered to be positive. The concentrations of eNOS, ET-1, sFlt, TNF-α, IL-4, IL-2, IL-6 and the coagulation-related genes, PAI-1 and t-PA, were detected using this method. Each experiment was performed three times.

### Scratch test

After digestion and counting, cells at logarithmic phase were inoculated into a 6-well plate (5 × 10^5^ cells/well) and maintained in a medium containing 10% FBS in a 5% CO_2_ incubator at 37°C for 24 h. When cell coverage reached or exceeded to 100%, the head of a 10 μl tip was scratched on the monolayer cells, the medium was aspirated, and the cells were maintained within FBS-free medium after washing in PBS. The cells were observed and photographed under an inverted microscope, then incubated in a 5% CO_2_ incubator at 37°C. The cell migration in each group was observed and photographed after 24 h and 48 h, respectively, and the scratched area was calculated using the Cell Profiler software. The healing rate of the scratched area which was calculated in the following method: (scratched area at 0 h—scratched area at different time points)/scratched area at 0 h × 100%. The experiment was performed three times.

### Cell Counting Kit-8 (CCK-8) assay

After digestion and centrifugation, HTR8/SVneo cells were planted into a 96-well plate (1 × 10^4^ cells/well), with 6 repeated wells in each group. The cells were transfected 24 h later. Another 4 to 6 hours later, the medium was changed into a 100 μL of complete medium. At 0 h, 12 h, 24 h and 48 h after transfection, the medium in the plate was fully aspirated, and 10 μL of water-soluble tetrazolium salt (WST) reagent and 90 μL of Dulbecco's modified eagle medium (DMEM) were added and mixed well to prevent the occurrence of bubbles. After incubation for 2 h in a 5% CO_2_ incubator at 37°C, the OD value was detected at a wavelength of 450 nm (reference wavelength was 650 nm). A figure was drawn with the x-axis representing time and the y-axis representing A450 nm. The experiment was repeated 3 times.

### 3-(4,5)-dimethylthiazol (-z-y1)-3,5-di- phenyltetrazolium bromide (MTT) assay

After digestion and centrifugation, HTR8/SVneo cells were planted into a 96-well plate (1 × 10^4^ cells/well), with 6 repeated wells in each group and the cells were transfected 24 h later. Four to six hours later, the medium was changed into a 100 μL of complete medium. At 0 h, 12 h, 24 h and 48 h after transfection, the medium in the plate was fully aspirated and a mixture of 10 μL of water-soluble tetrazolium salt (WST) reagent and 90 μL of DMEM were added. After incubation in a 5% CO_2_ incubator at 37°C for 2 h, the OD value at a wavelength of 450 nm was detected. In the blank control group, HTR8/SVneo cells were normally cultured. Cell cytotoxicity (%) = (OD260 in the blank control − OD260 in the exprimental group) × 100%. Each experiment was performed three times.

### Flow cytometry

After digestion and centrifugation, the HTR8/SVneo cells were planted into a 6-well plate (1 × 10^5^ cells/well) and the cells were routinely transfected. Forty-eight hours later, the cells were digested by 0.25% trypsin without ethylene diamine tetra acetic acid (EDTA). The digestion was observed under an inverted microscope (IX83, Olympus, Tokyo, Japan). To prevent digestion damage to HRT-8/SVneo cells, after 2 to 3 min, the digestion was terminated by adding complete medium when most cells became round. Afterwards, the cell suspension was moved into an eppendorf (EP) tube, and the cells were washed by PBS then centrifuged at 1500 rpm for 5 min, and then washed with PBS twice. Later, the cells were resuspended with 500 μL of binding buffer which reacted with 1 μL of Annexin V-PE and 5 μL of 7-AAD staining solution for 10 min. Within an hour, flow cytometry was used to detect cell apoptosis rate and reactive oxygen species (ROS) level in mitochondria at an excitation wavelength of 488 nm and an emission wavelength of 530 nm. The cell apoptosis rate was calculated by Annexin V + /PI cells/total cells. The experiment was performed three times.

### Establishment of the mouse models of PE and grouping

Phosphatidylcholine (PC) (80%) and phosphatidylserine (PS) (20%) (Sigma-Aldrich Chemical Company, St Louis MO, USA) were dissolved together in chloroform/methanol (V/V = 95.5) to an final concentration of 50 mg/mL. After drying with nitrogen, they were dissolved into a buffer solution (pH7.4) containing 0.05 mol/L Tris-HCL and 0.1 mol/L NaCl to a final concentration of 10 mg/mL. Under an ice-water bath at 4°C, the solution was filtered from a cellulose membrane with an aperture of 450 μm. Then, micelles witha diameter less than 300 μm were prepared and stored at a 4°C. Adult ICR (Institute of Cancer Research, USA) mice (26–30 g) purchased from the Animal Center of Nanjing Medical University (Nanjing, China), of which 40 female and 20 male mice, were availably fed with water and feed at 24°C in a pathogen free (SPF) grade labrotory with a constant temperature and humidity environment under 12-h light/dark cycle, and then placed into cages at a ratio of 2: 1. At 8: 00 the next day, smear examinations of vaginal suppository and vaginal secretion of female mice were performed. If the white vaginal suppository and the sperm in the secretion exhibited a positive result, the mouse was diagnosed as pregnant, and these pregnant mice were fed in cages with a record of day 0.5 of pregnancy. From 5.5^th^ day, 20 pregnant mice were randomly selected and injected with 0.1 mL PS/PC (1 mg/d) in the cauda vein every morning until day 16.5. This procedure established the mouse models of PE [13]. Another 10 pregnant mice were were treated with normal saline instead of PS/PC, with the same treatment procedure. The other 10 female mice were reserved for further use. All mice were randomly divided into normal pregnancy (NP, n = 10), preeclampsia (PE, n = 10) and preeclampsia + hAAT (PE + AAT, n = 10) groups, with their actions observed at 8–10 am everyday. Two days after model construction, the 10 mice in the PE + AAT group were injected with 200 μL PBS containing lentivirus-mediated AAT over-expression plasmid. And 17.5^th^ day after pregnancy, the mice were executed by cervical dislocation, followed by dissection for placental tissues among which some were soaked in 10% formaldehyde solution to fix for pathological section, which were then sliced by routine paraffin with a thickness of 4–5 μm. All experiments in this study were approved by the Ethics Committee of Wuxi Matemal and Child Health Hospital Affiliated to Nanjing Medical University.

### Blood pressure and urine protein measurements

The tail-cuff method was implemented to measure the systolic blood pressure (SBP) of the mice the 0.5^th^, 13.5^th^ and 16.5^th^ day after pregancy. Until the mice clam down, their blood pressures were measured in every successive 5 min, with the average as the confirmed blood pressure. And at the 16.5^th^ day, their urine proteins were detected. After placement in a metabolic cage at 9:00 P.M., the mice were fasted 12 h before urinary protein measurement, where water was freely available. At 9: 00 PM the next day, urine samples were collected in dry and sterile ampoules. After being filtered, the urine protein was measured.

### Determination of oxidative stress-related indexes

Cell and tissue homogenates in each group were extracted. The activity of SOD and the contents of MDA, H_2_O_2_ and GSH-PX were detected (Nanjing Institute of Jiancheng Biological Engineering, Nanjing, China; the experiment was conducted according to the instructions).

### Immunohistochemistry (IHC)

Expressions of AAT and p38MAPK in placental tissues were measured by a two-step method. The 10% formalin-fixed tissues were embedded in paraffin and sliced into 4-μm continuous sections, with the addition of diluted rabbit anti-mouse AAT primary antibody and p38MAPK polyclonal antibody (Beijing Bioss Biological Technology Co., Ltd., Beijing, China). Then, the slices were incubated in a 37°C incubatorfor 2 h and washed with PBS for 3 times (5 min/time). Afterwards, reagent I, reinforcing reagent, and reagent II were added into the slices in turn, and put inside an incubator for 20 min, respectively. Then samples were washed by PBS again for 3 times (5 min/time), followed by addition of 1 mL of distilled water was added into EP tube and biotin-labeled second antibody. Coloration was performed at room temperature and stopped with running water. After counterstaining by hematoxylin, redehydration by gradient ethanol and transparency by dimethylbenzene, the slices were mounted in neutral balsam and finally observed under a microscope, with IgG as a negative control. AAT was mainly expressed in the membrane, cytoplasm and intercellular substance, while p38MAPK was mainly expressed in the cytoplasm. Immunohistochemistry standard: staining sections were observed by at least two doctors pathologically in a double-blind fashion and the positive rate was described as stained cells/total cells.

### Statistical analysis

Commercial statistical analysis software was used for statistical analysis (SPSS 21.0; Inc., Chicago, IL, USA). Enumeration data were expressed as ratios or percentages, while the differences between groups were compared using the chi-square test. Measurement data were expressed as the mean ± standard deviation (SD), and the means between two groups were compared using *t*-test. One-way analysis of variance (ANOVA) was used for comparisons among multiple groups, before which homogeneity of variance was performed. A pairwise comparison of means among multiple groups was performed using least significant difference (LSD) method. A *P* < 0.05 was considered to indicate a statistically significant difference.

## Results

### Expressions of AAT, p-p38MAPK, STAT1 and ATF2 in HRT-8/SVneo cells among five groups

**Fig 1:**
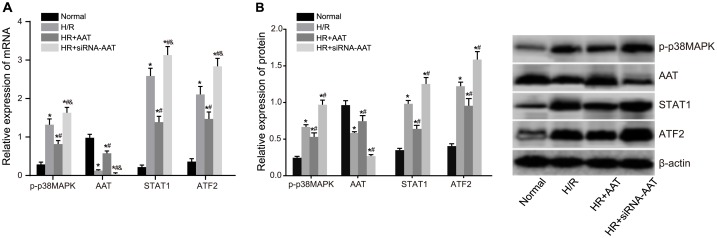
The protein expressions of AAT, p-p38MAPK, STAT1 and ATF2 in the normal, H/R, HR + AAT and HR + siRNA-AAT groups detected by Western blotting. Note: A, Western blotting image of protein expressions of AAT, p-p38MAPK, STAT1 and ATF2; B, histogram of protein expressions of AAT, p-p38MAPK, STAT1 and ATF2; *, *P* < 0.05 compared to the normal group; ^#^, *P* < 0.05 compared to the H/R group; ^*&*^, *P* < 0.05 compared to the HR + AAT group; normal, normal culture of HTR8/SVneo cells; H/R, 2 h of hypoxia and 4 h of reoxygenation of HTR8/SVneo cells; HR + AAT, 2 h of hypoxia and 4 h of reoxygenation of HTR8/SVneo cells after transfection with AAT over-expression plasmid; HR + siRNA-AAT, 2 h of hypoxia and 4 h of reoxygenation of HTR8/SVneo cells after transfection with siRNA-AAT overexpression plasmid; AAT, alpha-1-antitrypsin; p38MAPK, p38 mitogen-activated protein kinase; STAT1, signal transducer and activator of transcription 1; ATF2, activating transcription factor 2.

The results of qRT-PCR and Western blotting were presented in Table 2 and Fig 1, respectively. Compared with the normal group, the H/R, HR + AAT and HR + siRNA-AAT groups had decreased expression of AAT but increased expressions of p-p38MAPK, STAT1 and ATF2 (all *P* < 0.05). Compared with the H/R group, the expression of AAT increased significantly and the expressions of p-p38MAPK, STAT1 and ATF2 decreased significantly in the HR + AAT group (all *P* < 0.05), while the HR + siRNA-AAT group had decreased expression of AAT and increased expressions of p-p38MAPK, STAT1 and ATF2 (all *P* < 0.05).

**Table 2:** Comparisons of the mRNA expression of p-p38MAPK, AAT, STAT1, ATF2 detected by qRT-PCR in the normal, H/R, HR + AAT and HR + siRNA-AAT groups.

Group	Normal	H/R	HR + AAT	HR + siRNA-AAT
p38MAPK	0.29 ± 0.06	1.32 ± 0.15*	0.82 ± 0.09*^#^	1.65 ± 0.14*^#^^&^
AAT	0.98 ± 0.09	0.13 ± 0.02*	0.58 ± 0.06*^#^	0.04 ± 0.01*^#^^&^
STAT1	0.22 ± 0.05	2.59 ± 0.31*	1.39 ± 0.15*^#^	3.13 ± 0.39*^#^^&^
ATF2	0.36 ± 0.08	2.11 ± 0.29*	1.47 ± 0.18*^#^	2.84 ± 0.29*^#^^&^

Note:

*, *P* < 0.05 compared to the normal group;

^#^, *P* < 0.05 compared to the H/R group;

^*&*^, *P* < 0.05 compared to the HR + AAT group; normal, normal culture of HTR8/SVneo cells; H/R, 2 h of hypoxia and 4 h of reoxygenation of HTR8/SVneo cells; HR + AAT, 2 h of hypoxia and 4 h of reoxygenation of HTR8/SVneo cells after transfection with AAT over-expression plasmid; HR + siRNA-AAT, 2 h of hypoxia and 4 h of reoxygenation of HTR8/SVneo cells after transfection with siRNA-AAT overexpression plasmid; AAT, alpha-1-antitrypsin; p38MAPK, p38 mitogen-activated protein kinase; STAT1, signal transducer and activator of transcription 1; ATF2, activating transcription factor 2; qRT-PCR, quantitative real-time polymerase chain reaction.

### Effects of ATT gene on the secretion and OS in HRT-8/SVneo cells among five groups

As shown in Table 3, compared with the normal group, the H/R, HR + AAT and HR + siRNA-AAT groups had decreased activity of SOD and GSH-Px but increased MDA and H_2_O_2_ (all *P* < 0.05). Compared with the H/R group, the MDA and H_2_O_2_ decreased significantly but the activity of SOD and GSH-Px increased significantly in the HR + AAT group (all *P* < 0.05), while the HR + siRNA-AAT group had increased MDA and H_2_O_2_ but reduced activity of SOD and GSH-Px (all *P* < 0.05). ELISA results were presented in Table 4. Compared with the normal group, the H/R, HR + AAT and HR + siRNA-AAT groups had increased expressions of eNOS, ET-1, sFlt, t-PA, PAI-1, TNF-α, IL-4, IL-2 and IL-6 (all *P* < 0.05). Compared with the H/R group, the expressions of eNOS, ET-1, sFlt, t-PA, PAI-1, TNF-α, IL-4, IL-2 and IL-6 decreased significantly in the HR + AAT group, while the above indexes in the HR + siRNA-AAT group were up-regulated significantly (all *P* < 0.05).

**Table 3:** Comparisons of the activity of GSH-PX, MDA, T-SOD and H_2_O_2_ in the normal, H/R, HR + AAT and HR + siRNA-AAT groups.

Group	Normal	H/R	HR + AAT	HR+siRNA-AAT
GSH-PX (U/mg)	49.72 ± 5.52	25.29 ± 3.65*	37.01 ± 5.12*^#^	11.83 ± 1.72*^#^^&^
MDA (nmol/mg)	1.01 ± 0.13	8.99 ± 0.87*	5.71 ± 0.76*^#^	13.98 ± 2.25*^#^^&^
T-SOD (U/mg)	19.25 ± 2.13	8.71 ± 1.92*	14.25 ± 1.21*^#^	3.28 ± 0.59*^#^^&^
H_2_O_2_ (nmol/mg)	1.56 ± 0.24	9.79 ± 0.68*	5.79 ± 0.69*^#^	14.33 ± 1.76*^#^^&^

Note:

*, *P* < 0.05 compared to the normal group;

^#^, *P* < 0.05 compared to the H/R group;

^*&*^, *P* < 0.05 compared to the HR + AAT group; normal, normal culture of HTR8/SVneo cells; H/R, 2 h of hypoxia and 4 h of reoxygenation of HTR8/SVneo cells; HR + AAT, 2 h of hypoxia and 4 h of reoxygenation of HTR8/SVneo cells after transfection with AAT over-expression plasmid; HR + siRNA-AAT, 2 h of hypoxia and 4 h of reoxygenation of HTR8/SVneo cells after transfection with siRNA-AAT overexpression plasmid; GSH-PX, glutathione peroxidase; MDA, malondialdehyde; T-SOD, total superoxide dismutase; ROS, reactive oxygen species;

**Table 4:** Comparisons of the expression of inflammatory cytokines and related coagulation factors detected by ELISA in the normal, H/R, HR + AAT and HR + siRNA-AAT groups.

Group	Normal	H/R	HR + AAT	HR + siRNA-AAT
ET-1 (ng/L)	52.38 ± 5.31	156.47 ± 10.86*	118.52 ± 12.21*^#^	181.33 ± 6.69*^#^^&^
sFlt (ng/L)	5.98 ± 1.01	43.29 ± 3.52*	27.51 ± 2.67*^#^	76.87 ± 4.19*^#^^&^
eNOS (ng/L)	3.25 ± 0.57	26.18 ± 2.365*	18.73 ± 1.9*^#^	60.35 ± 1.16*^#^^&^
PAI-1 (ng/L)	11.26 ± 1.29	65.72 ± 7.59*	46.12 ± 6.85*^#^	99.12 ± 5.26*^#^^&^
TNF-α (ng/L)	11.98 ± 3.15	35.32 ± 3.85*	22.89 ± 3.22*^#^	46.01 ± 4.37*^#^^&^
IL-4 (ng/L)	125.06 ± 10.98	214.87 ± 9.35*	171.62 ± 12.59*^#^	259.97 ± 8.53*^#^^&^
IL-2 (ng/L)	8.95 ± 4.28	30.92 ± 5.01*	21.68 ± 7.35*^#^	41.31 ± 5.98*^#^^&^
IL-6 (ng/L)	88.42 ± 7.36	139.73 ± 5.99*	112.31 ± 8.19*^#^	185.15 ± 5.26*^#^^&^
t-PA (ng/L)	8.95 ± 1.25	31.32 ± 3.18*	19.52 ± 2.99*^#^	47.83 ± 1.95*^#^^&^

Note:

*, *P* < 0.05 compared to the normal group;

^#^, *P* < 0.05 compared to the H/R group;

^*&*^, *P* < 0.05 compared to the HR + AAT group; normal, normal culture of HTR8/SVneo cells; H/R, 2 h of hypoxia and 4 h of reoxygenation of HTR8/SVneo cells; HR + AAT, 2 h of hypoxia and 4 h of reoxygenation of HTR8/SVneo cells after transfection with AAT over-expression plasmid; HR + siRNA-AAT, 2 h of hypoxia and 4 h of reoxygenation of HTR8/SVneo cells after transfection with siRNA-AAT overexpression plasmid; ELISA, enzyme-linked immunosorbent assay; ET-1, endothelin-1; sFlt, soluble fms-like tyrosine kinase; eNOS, endothelial nitric oxide synthase; PAI-1, plasminogen activator Inhibitor-1; TNF-α, tumor necrosis factor-α; IL-4, interleukin-4; IL-2, interleukin-2; IL-6, interleukin-6; t-PA, tissue-type plasminogen activator.

### Effects of ATT gene on the proliferation, migration and apoptosis of HRT-8/SVneo cells among five groups

**Fig 2:**
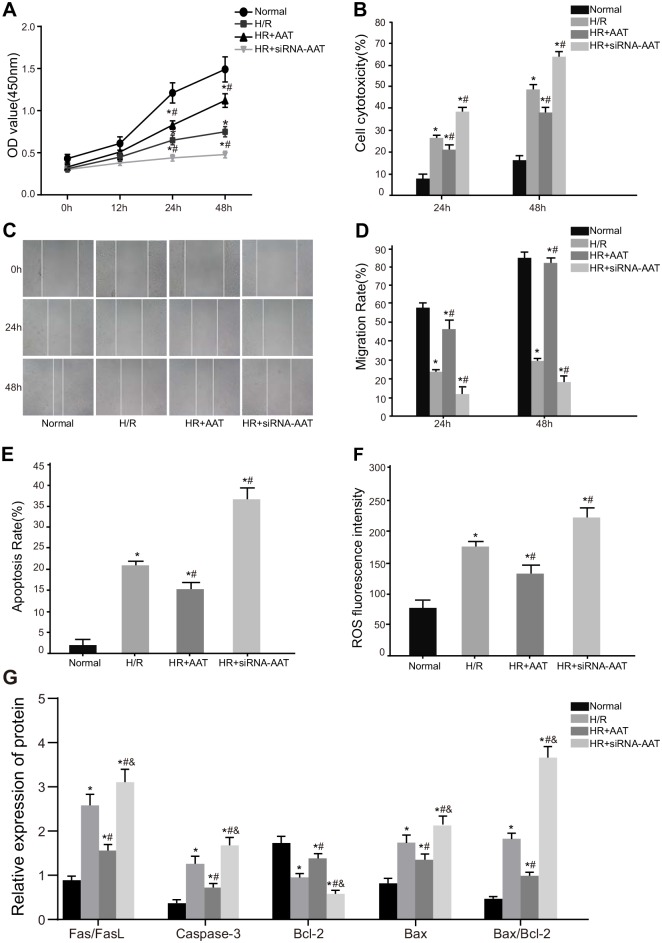
Cell proliferation, migration and apoptosis of HTR8/SVneo cells in the normal, H/R, HR + AAT and HR + siRNA-AAT groups. Note: A, comparison of OD value among the normal, H/R, HR + AAT and HR + siRNA-AAT groups; B, comparison of cell cytotoxicity among the normal, H/R, HR + AAT and HR + siRNA-AAT groups; C, comparison of cell migration distance among the normal, H/R, HR + AAT and HR + siRNA-AAT groups; D, comparison of cell migration rate among the normal, H/R, HR + AAT and HR + siRNA-AAT groups; E, comparison of cell apoptosis rate among the normal, H/R, HR + AAT and HR + siRNA-AAT groups; F, comparison of apoptosis-related protein expressions among the normal, H/R, HR + AAT and HR + siRNA-AAT groups; G, comparison of ROS fluorescence intensity among the normal, H/R, HR + AAT and HR + siRNA-AAT groups; *, *P* < 0.05 compared to the normol group; ^#^, *P* < 0.05 compared to the H/R group; ^*&*^, *P* < 0.05 compared to the HR + AAT group; normal, normal culture of HTR8/SVneo cells; H/R, 2 h of hypoxia and 4 h of reoxygenation of HTR8/SVneo cells; HR + AAT, 2 h of hypoxia and 4 h of reoxygenation of HTR8/SVneo cells after transfection with AAT over-expression plasmid; HR + siRNA-AAT, 2 h of hypoxia and 4 h of reoxygenation of HTR8/SVneo cells after transfection with siRNA-AAT overexpression plasmid; ROS, reactive oxygen species; AAT, alpha-1-antitrypsin.

The detection of the proliferation of HRT-8/SVneo cells at 0 h, 12 h, 24 h and 48 h after transfection showed that after H/R, the H/R, HR + AAT and HR + siRNA-AAT groups had decreased cell proliferation and migration but increased cell cytotoxicity at 24 h and 48 h compared with the normal group (all *P* < 0.05). Compared with the H/R group, the cell proliferation and migration increased significantly but cell cytotoxicity decreased significantly in the HR + AAT group (all *P* < 0.05), while the cell proliferation and migration were reduced and cell cytotoxicity was raised in the HR + siRNA-AAT group (all *P* < 0.05) (Fig 2A–2D). Results of flow cytometry assay and Western blotting showed that compared with the normal group, the H/R, HR + AAT and HR + siRNA-AAT groups had increased cell apoptosis rate, ROS, Fas/FasL, Caspase-3, Bax and Bax/Bcl-2 but decreased Bcl-2 (all *P* < 0.05). Compared with the H/R group, the cell apoptosis rate, ROS, Fas/FasL, Caspase-3, Bax and Bax/Bcl-2 decreased significantly but Bcl-2 increased significantly in the HR + AAT group (all *P* < 0.05), while the increased cell apoptosis rate, ROS, Fas/FasL, Caspase-3, Bax and Bax/Bcl-2 but reduced Bcl-2 were observed in the HR + siRNA-AAT (all *P* < 0.05) (Fig 2E–2G).

### Comparisons of blood pressure and urine protein in mice among the NP, PE, and PE + AAT groups

The blood pressure and urine protein expressions were presented in Table 5. In comparison with the NP group, in the PE group, blood pressure increased significantly on the 13.5^th^ and 16.5^th^ day and urine protein increased significantly on the 16.5^th^ day (all *P* < 0.05). As the main symptoms of PE are high blood pressure and urine protein, the results suggested PS/PC treatment mimized the symptoms of PE, therefore indicating mouse models were well-established.

**Table 5:** Comparisons of blood pressure and urine protein expressions in mice among NP, PE, and PE + AAT groups.

Group	Blood pressure (mmHg)			Urine protein (mg/L)
	gd0.5	gd13.5	gd16.5	gd16.5
NP	102.67 ± 5.11	104.24 ± 5.38	101.69 ± 5.65	178.61 ± 42.37
PE	108.24 ± 4.23	131.28 ± 6.23*	134.28 ± 6.89*	716.18 ± 120.35*
PE + AAT	105.24 ± 3.53	116.35 ± 4.35^#^	120.35 ± 4.53^#^	348.78 ± 90.36^#^

Note: *P* < 0.05 compared to the PE + AAT group; '

^#^, *P* < 0.05 compared to the NP group; NP, normal pregnancy; PE, preeclampsia; AAT, alpha-1-antitrypsin.

### Expressions of AAT and p-p38MAPK in placental tissues among the NP, PE, and PE + AAT groups

**Fig 3:**
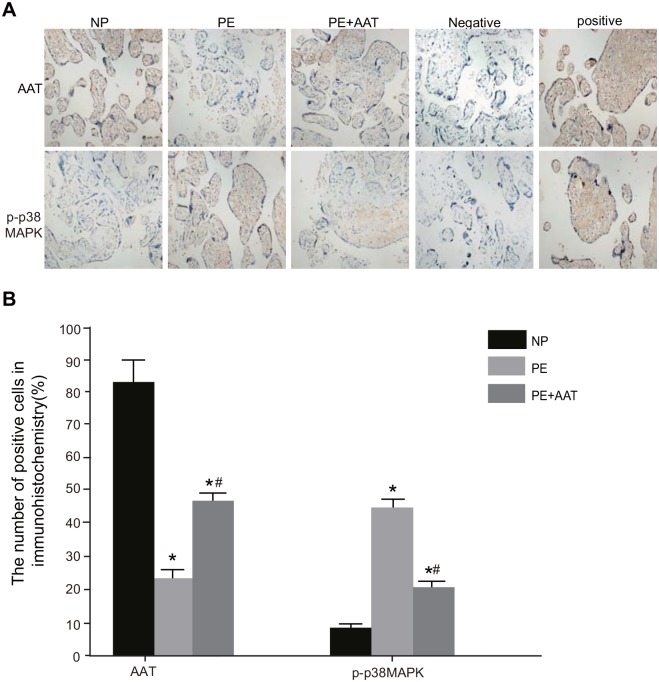
AAT and p-p38MAPK expressions in placental tissues of mice in the NP, PE and PE + AAT groups detected by immunohistochemistry. Note: A, expressions of AAT and p-p38MAPK in placental tissues detected by immunohistochemistry in the NP, PE and PE + AAT groups; B, the number of cells with AAT and p-p38MAPK expressions detected by immunohistochemistry in the NP, PE and PE + AAT groups; AAT, alpha-1-antitrypsin; p38MAPK, p38 mitogen-activated protein kinase; NP, normal pregnancy; PE, preeclampsia; *, *P* < 0.05 compared to the NP group; ^#^, *P* < 0.05 compared to the PE group.

Showed by immunohistochemistry (Fig 3), AAT was mainly expressed in the membrane, cytoplasm and intercellular substance of placental trophoblastic cells. In addition, all three groups showed AAT expressions. Among the three groups, the NP group had largest positively-expressed cell number and drained area of cytoplasm, then followed by the PE + AAT group and the PE group (all *P* < 0.05). The p-p38MAPK was mainly expressed in the cytoplasm and a few nuclei of placental trophoblastic cells. Among the three groups, the PE group had higher expression of p-p38MAPK than the NP group, but the expression of p-p38MAPK in the PE + AAT group was higher than the NP group but lower than the PE group (all *P* < 0.05).

### Comparisons of the secretion and OS indexes between placental tissues in PE and normal placental tissues

**Fig 4:**
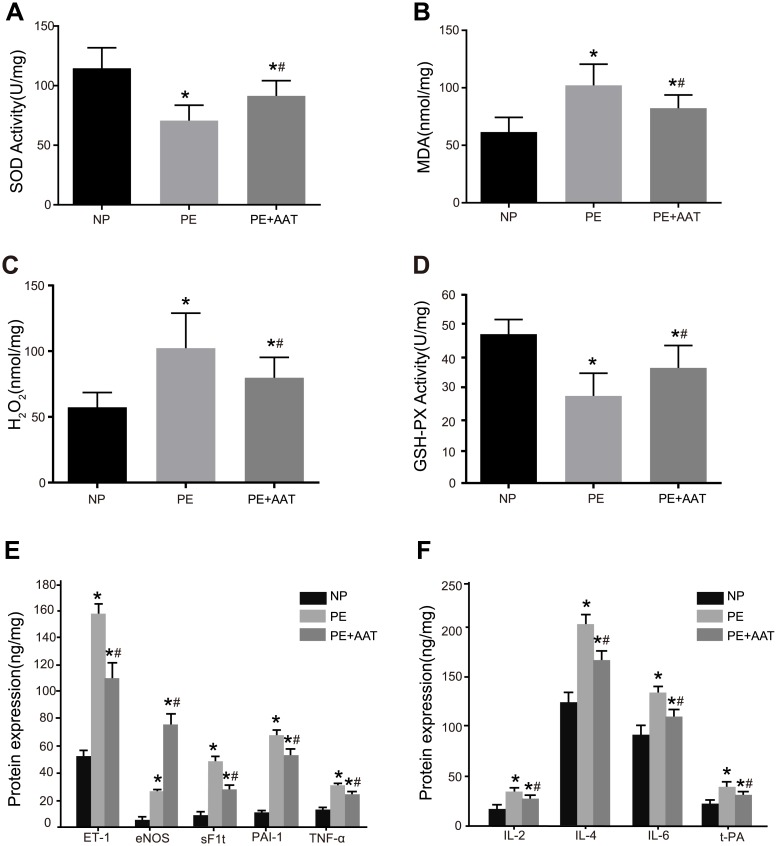
The secretion and OS indexes in placental tissues of mice in the NP, PE and PE + AAT groups. Note: A, SOD activity detected by ELISA; B, MDA detected by ELISA; C, H_2_O_2_ detected by ELISA; D, GSH-PX activity detected by ELISA; E, expressions of eNOS, ET-1, sFlt, PAI-1 and TNF-α detected by ELISA; F, expressions of t-PA, IL-4, IL-2 and IL-6 detected by ELISA; *, *P* < 0.05 compared to the PE + AAT group; ^#^, *P* < 0.05 compared to the NP group; ^*@*^, *P* < 0.05 compared to the PE group; OS, oxidative stress; NP, normal pregnancy; PE, preeclampsia; SOD, superoxide dismutase; GSH-PX, glutathione peroxidase; MDA, methylenedioxyamphetamine; ET-1, endothelin-1; sFlt, soluble fms-like tyrosine kinase; eNOS, endothelial nitric oxide synthase; PAI-1, plasminogen activator Inhibitor-1; TNF-α, tumor necrosis factor-α; IL-4, interleukin-4; IL-2, interleukin-2; IL-6, interleukin-6; t-PA, tissue-type plasminogen activator; ELISA, enzyme-linked immunosorbent assay.

Compared with the NP group, the placental tissues in the PE and PE + AAT groups had decreased activity of SOD and GSH-Px but increased MDA and H_2_O_2_ contents and expressions of eNOS, ET-1, sFlt, t-PA, PAI-1, TNF-α, IL-4, IL-2 and IL-6 (all *P* < 0.05). Compared with the PE group, the MDA and H_2_O_2_ contents and expressions of eNOS, ET-1, sFlt, t-PA, PAI-1, TNF-α, IL-4, IL-2 and IL-6 decreased significantly but the activity of SOD and GSH-Px increased significantly in the PE + AAT group (all *P* < 0.05) (Fig 4).

### Comparisons of apoptosis-related indexes in placental tissues in PE among the NP, PE, and PE + AAT groups

**Fig 5:**
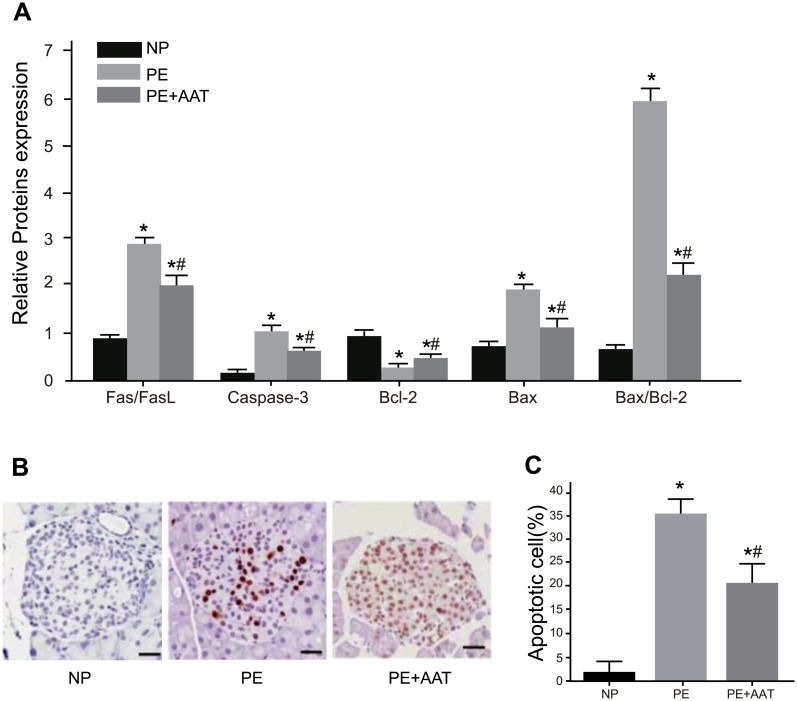
Comparisons of apoptosis-related protein expressions and apoptotic cell number among among NP, PE and PE + AAT groups; A, expressions of Fas/FasL, Caspase-3, Bax, Bax/Bcl-2 and Bcl-2 detected by Western blotting; B, cell apoptosis detected by TUNEL; C, apoptotic cell number in the NP, PE and PE + AAT groups; *, *P* < 0.05 compared to the PE + AAT group; ^#^, *P* < 0.05 compared to the NP group; NP, normal pregnancy; PE, preeclampsia; TUNEL, terminal deoxynucleotidyl transferase-mediated dUTP nick end labeling.

As shown in Fig 5, compared with the NP group, the PE and PE + AAT groups had decreased expressions of Bcl-2 but increased Fas/FasL, Caspase-3, Bax and Bax/Bcl-2 in placental tissues of mice (all *P* < 0.05). Compared with the PE group, the expressions of Fas/FasL, Caspase-3, Bax and Bax/Bcl-2 decreased significantly but Bcl-2 increased significantly in the PE + AAT group (all *P* < 0.05).

## Discussion

Although many studies of PE demonstrate that poor placentation can cause oxidative stress, apoptosis, endoplasmic reticulum stress and endothelial dysfunction of the placenta [14–17], there are few data that elucidate the underlying mechanism of oxidative stress-mediated signaling pathways in terms of the development of PE. Our study aims to investigate the roles of AAT and the p38MAPK signaling pathway in OS during PE. The data revealed that AAT could reduce OS, promote proliferation and migration and suppress apoptosis of HTR-8/SVneo cells via inhibiting the p38MAPK signaling pathway in PE.

In our study, the mice in PE group have higher blood pressure and proteinuria, increased MDA and H_2_O_2_ levels but decreased SOD activity compared to the mice with normal pregnancy. PE is a pregnancy-specific syndrome characterized by hypertension and proteinuria [18]. Consistent with our study, Bosco et al. detected a lower activity of SOD in PE placentas compared with normal placentas [19]; A previous study also confirmed that H_2_O_2_ and MDA levels were also detected significantly increased in preeclamptic placentas than in normal ones [20, 21].

Our findingsuggest that AAT overexpression decreased the MDA level and elevated the activities of both SOD and GSH-Px damaged by H/R inury, which is further confirmed by the evidence that the PE mice with AAT overexpression had higher SOD activity but lower MDA or H_2_O_2_ levels in the placental tissues as compared to the PE mice. Oxidative stress of the placenta is a leading risk factor, leading to endothelial dysfunction by direct actions on the vasculature [22]. Various important antioxidants, such as SOD and glutathione peroxidase (GPx) (which is able to protectthe vasculature from ROS damage and maintain the vascular function at normal levels), are remarkably reduced in the maternal circulation stage of women with PE [23]. Through a dose- and time-dependent manner, exogenous AAT can alleviate the damage caused by H/R, which implies the cytoprotective role of AAT in vascular endothelial cells [24]. The work of Feng et al. indicates that AAT injection plays an anti-oxidative stress role by facilitating a significant reduction of PE mediated-elevation of MDA and ROS and an increase in the levels of GPx and SOD with increased AAT dosage [12], which is consistent with our results.

Additionally, compared with the normal HTR-8/SVneo cells, the AAT expression was reduced, while p38MAPK, STAT1 and ATF2 expressions were increased in the HTR-8/SVneo cells after H/R treatment, suggesting that AAT and OS oppose each other and that oxidative stress can promote the p38MAPK signaling pathway. Similarly, Cindrova-Davies et al. discovered that HR-induced oxidative stress could activate the p38MAPK signaling pathway at the onset of PE [7]. In addition, compared with the normal HTR-8/SVneo cells, the HTR-8/SVneo cells with AAT overexpression have reduced the expressions of p38MAPK, STAT1 and ATF2, indicating that AAT may participate in OS via down-regulation of the p38MAPK signaling pathway, which is further confirmed by in vivo experiment that the placental tissues of PE mice with AAT expression have weaker p-p38MAPK expression than the PE mice.

Our study also revealed that after H/R treatment, the expressions of eNOS, ET-1, sFlt, coagulation-related genes (PAI-1 and t-PA), and inflammatory factors (TNF-α, IL-4, IL-2 and IL-6) is elevated. An elevated eNOS level increases NO production, which may be an adaptive response to the increased resistance as well as poor perfusion in the pathological pregnancies [25]. Additonally, H/R intervention causes p38 activation and ultimately leads to an increase in sFlt-1 secretion in human umbilical vein endothelial cells [26]. It has been reported that increased circulating ET-1 is associated with PE [27] and that PAI-1 levels were found up-regulated in patients with PE [28], which is related to enhance inflammatory reactions [29]. A previous study supported our results that AAT could inhibit cell secretion and the expressions of secretion function-related genes (eNOS, ET-1, sFlt), coagulation-related factors, and inflammatory factors by regulating the p38MAPK signaling pathway. It was found that eNOS and sFltwas reduced when AAT is increased [22]. AAT can also inhibit the proteinases and restrain the ectatic process, which hasbeneficial effects on the balance of the coagulation system [30]. As Jonigk et al. suggested that AAT could possesse anti-inflammatory as well as immunomodulatory properties [31]. Evidence has shown that oxidative stress can activate the p38MAPK signaling pathway, which results in a maternal inflammatory reaction manifested in endothelial dysfunction and the symptoms of PE [15]. As reflected in our study, the addition of PMA (an agonist of p38MAPK) recovers the cell secretion function.

It has been found in our study that AAT overexpression can increase the migration and proliferation abilities of the HTR-8/SVneo cells after H/R damage. Feng et al. have also indicated that overexpression of AAT decreases cell apoptosis and increases cell proliferation through the inhibition of the p38 signaling pathway and oxidative stress [24]. It was also revealed in our study that AAT can reduce cell apoptosis by regulating the p38MAPK signaling. In the H/R treated HTR-8/SVneo cells, elevated AAT expression can decrease cell apoptosis and apoptosis factors (Fas/FasL and caspase) as well as increase anti-apoptosis factors (Bcl-2). In PE, the apoptosis rate was increased because of oxidative stress and hypoxia reperfusion injury, and Bcl-2 protein can especially cause H/R-induced apoptosis in the placenta [32]. That may be due to p38 inhibition, which can provide protective effects on H/R-exposed human umbilical vein endothelial cells by suppressing oxidative stress and inhibiting apoptosis [26].

In conclusion, our study suggests that AAT could suppress oxidative stress, promote proliferation and migration and suppress apoptosis of HRT-8/SVneo cells and improve PE via inactivating the p38MAPK signaling pathway, which may serve as a novel therapeutic strategy applicable to the management of PE. Considering that the experiments *in vivo* were performed in mice instead of human beings, the data might not be convincing enough to confirm our findings. Therefore, our results shall be further verified and enriched by future studies to enhance the power of our results.
